# Essential Oil from *Arnica Montana* L. Achenes: Chemical Characteristics and Anticancer Activity

**DOI:** 10.3390/molecules24224158

**Published:** 2019-11-16

**Authors:** Danuta Sugier, Piotr Sugier, Joanna Jakubowicz-Gil, Krystyna Winiarczyk, Radosław Kowalski

**Affiliations:** 1 Department of Industrial and Medicinal Plants, University of Life Sciences in Lublin, 15 Akademicka Street, 20-950 Lublin, Poland; danuta.sugier@up.lublin.pl; 2 Department of Botany, Mycology and Ecology, Institute of Biological Sciences, Maria Curie-Skłodowska University, 19 Akademicka Street, 20-033 Lublin, Poland; 3 Department of Functional Anatomy and Cytobiology, Institute of Biological Sciences, Maria Curie-Skłodowska University, 19 Akademicka Street, 20-033 Lublin, Poland; jjgil@poczta.umcs.lublin.pl; 4 Department of Cell Biology, Institute of Biological Sciences, Maria Curie-Skłodowska University, 19 Akademicka Street, 20-033 Lublin, Poland; krystyna.winiarczyk@poczta.umcs.lublin.pl; 5 Department of Analysis and Evaluation of Food Quality, University of Life Sciences in Lublin, 8 Skromna Street, 20-704 Lublin, Poland; radoslaw.kowalski@up.lublin.pl

**Keywords:** *Arnica montana* L., achenes, essential oil composition, anticancer activity activity, MOGGCCM and T98G cell lines

## Abstract

Mountain arnica *Arnica montana* L. is a source of several metabolite classes with diverse biological activities. The chemical composition of essential oil and its major volatile components in arnica may vary depending on the geographical region, environmental factors, and plant organ. The objective of this study was to characterize the chemical composition of essential oil derived from *A. montana* achenes and to investigate its effect on induction of apoptosis and autophagy in human anaplastic astrocytoma MOGGCCM and glioblastoma multiforme T98G cell lines. The chemical composition of essential oil extracted from the achenes was examined with the use of Gas Chromatography–Mass Spectrometry GC-MS. Only 16 components of the essential oil obtained from the achenes of 3-year-old plants and 18 components in the essential oil obtained from the achenes of 4-year-old plants constituted ca. 94.14% and 96.38% of the total EO content, respectively. The main components in the EO from the arnica achenes were 2,5-dimethoxy-p-cymene (39.54 and 44.65%), cumene (13.24 and 10.71%), thymol methyl ether (8.66 and 8.63%), 2,6-diisopropylanisole (8.55 and 8.41%), decanal (7.31 and 6.28%), and 1,2,2,3-tetramethylcyclopent-3-enol (4.33 and 2.94%) in the 3- and 4-year-old plants, respectively. The essential oils were found to exert an anticancer effect by induction of cell death in anaplastic astrocytoma and glioblastoma multiforme cells. The induction of apoptosis at a level of 25.7–32.7% facilitates the use of this secondary metabolite in further studies focused on the development of glioma therapy in the future. Probably, this component plays a key role in the anticancer activity against the MOGGCCM and T98G cell lines. The present study is the first report on the composition and anticancer activities of essential oil from *A. montana* achenes, and further studies are required to explore its potential for future medicinal purposes.

## 1. Introduction

*Arnica montana* L. is a source of several active compounds (sesquiterpene lactones, flavonoids, terpenoids, phenolic acids, and essential oils) exhibiting antibacterial, antifungal, antiseptic, anti-inflammatory, antiradical, antisclerotic, and antioxidant activities [1,2,3,4,5,6,7,8,9]. The chemical composition of essential oil (EO) and its major volatile components in arnica may vary depending on the geographical region and environmental factors [4,5,6,7,9,10,11]. Essential oils extracted from different organs of many specimens coming from different regions of Europe are composed mainly of sesquiterpene hydrocarbons (e.g., E-caryophyllene, germacrene D, α-humulene, bicyclogermacrene), oxygenated monoterpenoids (e.g., 1,8-cineole, linalool), oxygenated sesquiterpenoids (e.g., caryophyllene oxide, α-cadinol), and phenyl derivative compounds (e.g., 2,5-dimethoxy-p-cymene, thymol methyl ether, p-methoxyheptanophenone, and 2,6-diisopropylanisole) [4,7,9,10,11,12]. As reported in the literature, EO extracted from such arnica organs as flower heads, rhizomes, plant roots, and hair roots may vary. This fact implies a very broad possibility of the use of the chemically different secondary metabolites. The seed yield and germination characteristics were studied during introduction of a wild arnica population into field conditions [6,13,14,15,16]. Seed dispersal was analyzed in terms of plant protection as well as restoration and development of endangered plant communities [17]. Our earlier study focused on the phenolic profiles and antioxidant abilities of achene extracts as novel sources of natural antioxidants [2], with a view to carrying out further investigations of chemical compounds contained in mountain arnica achenes. This study was conducted to determine a new quality of secondary metabolites in, e.g., *A. montana*, which is a very interesting and valuable pharmacopeal plant species [6,7,10]. Achenes are an alternative source of biological substances besides flower heads, rhizomes, roots, and herb.

Cancer is a growing global problem. In a report on the global burden of cancer, it was estimated that there would be 18.1 million new cases and 9.6 million cancer deaths worldwide in 2018. In the case of the brain and nervous system, there were 296,851 new cases and over 81% of death cases [18]. Therefore, many alternative treatments and therapies based on plants have been explored, which is especially important for patients that do not tolerate extreme side effects. Although the research on the application of EOs as anticancer therapeutic agents is relatively new, approximately half of conventional chemotherapy agents have plant origin, with roughly 25% directly derived from plants and 25% being chemically modified versions of phytoproducts [19]. Plants have been analyzed to identify their anticancer properties and chemically characterized to reveal the presence of many bioactive compounds, e.g., polyphenols, taxols, brassinosteroids, etc. [20]. The major group of malignant gliomas is represented by anaplastic astrocytoma (AA, WHO grade III) and glioblastoma multiforme (GBM, WHO grade IV). Despite the tremendous efforts in improvement of therapeutic measures, such as surgery, radiotherapy, and chemotherapy, the clinical outcome of gliomas remains dismaying [21,22]. Various types of malignancies such as gliomas are reported to decrease after treatment with plant essential oils [23]. Hence, such molecules are assumed to have potential anticancer activities that are useful in prevention and therapeutic strategies [20,24]. Therefore, there is an urgent need for searching for new substances, e.g., essential oils, to elucidate the molecular basis of malignant progression of gliomas [25,26,27,28,29,30,31,32].

Natural products and their derivatives are important sources of novel therapeutic molecules [33]. EOs have been applied in traditional medicine systems since ancient times in human history. Researchers from all over the world are trying to characterize the range of biological properties of EO, which includes antimicrobial, antiviral, antimutagenic, anticancer, antioxidant, anti-inflammatory, immunomodulatory, and antiprotozoal activities [20,34,35,36,37,38,39]. The interest in medicinal plants is continuously growing due to the increasing human demand [40]; therefore, new combinations of EO components can exhibit new properties and activities that can be used in medicine, pharmacy, or cosmetic industry in the future. In the case of *A. montana*, the knowledge of the chemical characteristics and biological activity of achenes is insufficient [2]. Therefore, the results presented in this paper fill this gap partially and provide information that can be the first step in studies of the anticancer activity of secondary metabolites from the mountain arnica. Therefore, the objective of this study was (i) to characterize the chemical composition of EO derived from *A. montana* achenes collected from 3-year-old and 4-year-old plants, and (ii) to investigate the effect of the analyzed EO on induction of apoptosis and autophagy in human anaplastic astrocytoma MOGGCCM and glioblastoma multiforme T98G cell lines.

## 2. Results

### 2.1. Chemical Characteristics of EO

The yield of the essential oil and the content of the components of EO obtained from the achenes of 3-year-old plants (EO-3) and EO obtained from the achenes of 4-year-old plants (EO-4) of mountain arnica are presented in Table 1. The content of EO was at a level of 0.167% and 0.145% v/w in the EO-3 and EO-4, respectively. There are slight differences in the chemical composition between the two samples. Only 16 components in the EO from the achenes collected from the 3-year-old specimens and 18 components in the EO from the achenes of the 4-year-old plants constituted ca. 94.14% and 96.38% of the total EO content, respectively. The studied EO was dominated by phenyl derivative constituents, which accounted for 48.09 and 53.06% in the EO-3 and EO-4, respectively (Table 1). Monoterpenes (23.77 and 20.49%) were the second major class of compounds. The other class of compounds was represented by sesquiterpenes (10.06 and 10.05%) and aliphatic aldehydes (7.31 and 6.28% in the EO-3 and EO-4, respectively). The main components in the EO from the arnica achenes were 2,5-dimethoxy-p-cymene (39.54 and 44.65%), cumene (13.24 and 10.71%), thymol methyl ether (8.66 and 8.63%), 2,6-diisopropylanisole (8.55 and 8.41%), decanal (7.31 and 6.28%), and 1,2,2,3-tetramethylcyclopent-3-enol (4.33 and 2.94%) in the 3- and 4-year-old plants, respectively. The concentration of such EO components as cumene, 1,2,2,3-tetramethylcyclopent-3-enol, α-pinene oxide, β-maaliene, E-α-bergamotene, and lippifoli-1(6)-en-5-one was higher in EO-3. In turn, higher concentrations of 7-epi-silphiperfol-5-ene, α-isocomene, 2,5-dimetoxy-p-cymene, E-caryophyllene, and caryophyllene oxide were determined in EO-4.

### 2.2. Anticancer Activity

**Figure 1:**
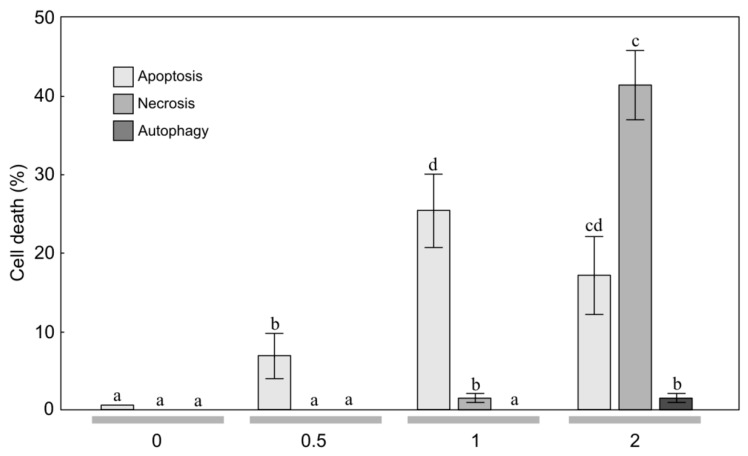
Level of apoptosis, necrosis, and autophagy induction in anaplastic astrocytoma MOGGCCM cells treated with the essential oil (concentration: 0, 0.5, 1, 2 μL/mL) from mountain arnica achenes collected from 3-year-old plants. The values designated by the different letters are significantly different (*p* = 0.05). (Tukey test, *p* < 0.05).

**Figure 2:**
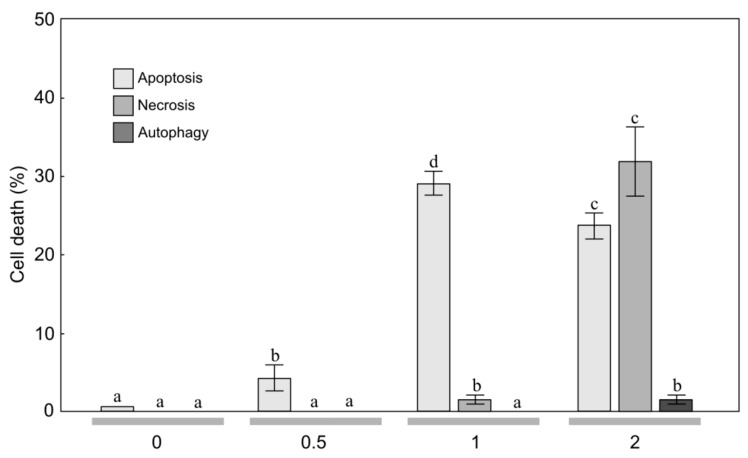
Level of apoptosis, necrosis, and autophagy induction in anaplastic astrocytoma MOGGCCM cells treated with the essential oil (concentration: 0, 0.5, 1, 2 μL/mL) from mountain arnica achenes collected from 4-year-old plants. The values designated by the different letters are significantly different (*p* = 0.05). (Tukey test, *p* < 0.05)

**Figure 3:**
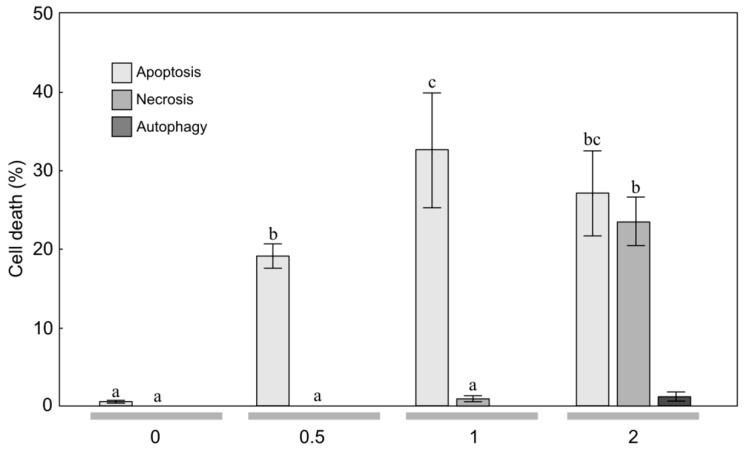
Level of apoptosis, necrosis, and autophagy induction in glioblastoma multiforme T98G cells treated with the essential oil (concentration: 0, 0.5, 1, 2 μL/mL) from mountain arnica achenes collected from 3-year-old plants. The values designated by the different letters are significantly different (*p* = 0.05). (Tukey test, *p* < 0.05).

**Figure 4:**
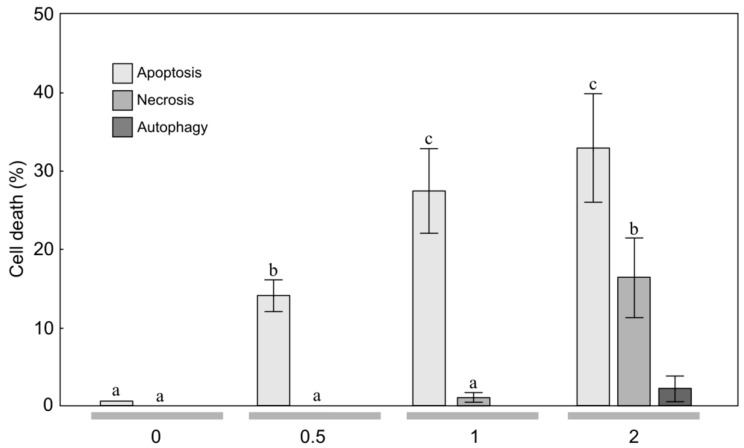
Level of apoptosis, necrosis, and autophagy induction in glioblastoma multiforme T98G cells treated with the essential oil (concentration: 0, 0.5, 1, 2 μL/mL) from mountain arnica achenes collected from 4-year-old plants. The values designated by the different letters are significantly different (*p* = 0.05). (Tukey test, *p* < 0.05).

To estimate the sensitivity of MOGGCCM and T98G cells to treatment with the EO from arnica achenes, a staining method with dyes specific for apoptosis, necrosis, and autophagy; i.e., Hoechst 33342, propidium iodide, and acridine orange, respectively, was employed (Figure 1, Figure 2, Figure 3 and Figure 4). The two-way ANOVA results showed a significant main effect of the EO concentration only on apoptosis, necrosis and autophagy of the MOGGCCM cells (F = 160.70, *p* < 0.001; F = 877.9, *p* < 0.001; F = 16.18, *p* < 0.001, respectively) and the T98G cells (F = 416.11, *p* < 0.001; F = 5.79, *p* < 0.01; F = 5.42, *p* < 0.01, respectively). In contrast, no statistically significant effect of the plant age (F = 0.75, *p* = 0.400; F = 0.51, *p* = 0.486; F = 0.14, *p* = 0.711, respectively) and the EO concentration/plant age interaction was confirmed (F = 1.7, *p* = 0.193; F = 0.68, *p* = 0.382; F = 0.17, *p* = 0.933, respectively).

The microscopic observations revealed that EO-3 added to the MOGGCCM culture medium at a concentration of 0.5 μL/mL exerted a considerable effect on induction of cell death (Figure 1). A significant 7.0% increase in the number of apoptotic cells under the influence of EO-3 was observed at a concentration of 0.5 μL/mL (Figure 1). A further increase in the EO concentration resulted in an increase in the level of apoptosis to 25.7%. Besides apoptosis, EO-3 initiated necrosis at a level of ca. 1.3% only. The EO concentration of 2 μL/mL caused a decrease in the percentage of apoptotic cells, but it was not statistically significant. However, despite the apoptosis occurring at this EO concentration, necrosis (41.7%) and autophagy (1.0%) was initiated.

The application of 0.5 μL/mL of EO-4 to the MOGGCCM culture medium had a considerable effect on cell death, i.e., a significant 4.0% increase in the number of apoptotic cells was observed at this concentration (Figure 2). A further increase in the EO concentration also resulted in a significant increase in the level of apoptosis to over 29.0%. The EO-4 concentration of 2 μL/mL caused a statistically significant decrease in the percentage of apoptotic cells (23.7%). However, despite the apoptosis occurring at this EO concentration, necrosis was initiated at a level 31.7%, which was higher than the level of apoptosis. It is worth underlining that both EO-3 and EO-4 did not initiate autophagy in the MOGGCCM culture medium (Figure 1 and Figure 2).

The microscopic observations showed that EO-3 applied at the concentration of 0.5 μL/mL to the T98G culture medium had a considerable effect on cell death (Figure 3). A significant 19.3% increase in the number of apoptotic cells under the influence of the EO was observed at the concentration of 0.5 μL mL (Figure 3). A further increase in the EO-3 concentration also resulted in a ca. 32.7% increase in the level of apoptosis. Besides apoptosis, EO initiated necrosis, but only at a level of ca. 0.3%. The EO concentration of 2 μL/mL caused a statistically significant decrease in the percentage of apoptotic cells to the level of 28.1%. However, despite the apoptosis occurring at this EO concentration, necrosis at a level exceeding the percentage of apoptotic cells (23.7%) was initiated.

The application of EO-4 to the T98G culture medium (Figure 4) caused a similar reaction to that described above (Figure 3). A significant 14.3% increase in the number of apoptotic cells was induced by EO-4 applied at the concentration of 0.5 μL/mL. A further increase in the EO concentration to 1 μL/mL also resulted in a ca. 27.7% increase in the level of apoptosis. In turn, the concentration of 2 μL/mL caused a gradual increase in the percentage of apoptotic cells to 33.0%; however, it was not statistically significant. Besides apoptosis, the EO initiated necrosis at a level 16.7%. It is worth underlining that both EO-3 and EO-4 did not cause autophagy in the T98G culture medium. Analogically to the MOGGCCM line (Figure 1 and Figure 2), the effect of EO-3 and EO-4 on autophagy induction in the T98G line was not significant (Figure 3 and Figure 4).

The IC50 analysis also revealed that the MOGGCCM cells were more sensitive to initiation of cell death after the treatment with the essential oils in comparison to the T98G cell line (Table 2). The analyzed essential oils had no effect on cell death initiation in normal fibroblasts (data not shown).

## 3. Discussion

Sixteen components in the EO-3 mountain arnica plants and 18 components in EO-4 constituted ca. 94.14% and 96.38% of the total oil content, respectively. The number and diversity of components in the EO from *A. montana* achenes was lower in comparison to EO from flower heads analyzed in a population from Lithuania [11]. In the present study, the number of constituents was by 34 lower than in EO from flower heads from Bosnia [45], by 42 lower than in EO from flower heads from Serbia [10], and by 24 lower than in EO from flower heads from Poland demonstrated in our previous study [7,9].

The main components in the EO from the arnica achenes were 2,5-dimethoxy-p-cymene (39.54 and 44.65%), cumene (13.24 and 10.71%), thymol methyl ether (8.66 and 8.63%), 2,6-diisopropylanisole (8.55 and 8.41%), decanal (7.31 and 6.28%), and 1,2,2,3-tetramethylcyclopent-3-enol (4.33 and 2.94%) in EO-3 and EO-4, respectively. This EO composition is most similar to the chemical composition of EOs from arnica rhizomes and roots reported by Pljevjakušić et al. [4]. 2,5-dimethoxy-p-cymene was demonstrated by the authors as the main component of EO from rhizomes (28.9–30.0%) and roots (37.9–40.6%). The other most abundant molecule in the rhizomes and roots was thymol methyl ether, with a concentration of 26.1% and 27.2%, respectively. In turn, Weremczuk-Jeżyna et al. [12] identified two main components in EO from hairy roots in vitro and plant roots of *Arnica montana*, i.e., 10-isobutyryloxy-8,9-didehydro-thymol isobutyrate and 10-isobutyryloxy-8,9-didehydro-thymol methyl ether. Interestingly, 2,5-dimethoxy-p-cymene, i.e., the dominant compound in the EO from the arnica achenes was not detected in the EO from the mountain arnica flower heads analyzed in our earlier studies [7,9]. However, the main components in the EO from the flower heads were E-caryophyllene, germacrene D, cumene, p-cymene, decanal, and caryophyllene oxide [9]. The EO profile in arnica achenes is similar to the EO profile in arnica rhizomes [4], but completely different from the EO profile in arnica flower heads. In the present study, phenyl derivatives were the most abundant group (48.09% and 53.06%), followed by monoterpenes (23.77% and 20.49%) and sesquiterpene (10.06% and 10.05%). However, the EO obtained from arnica flower heads was characterized by dominance of sesquiterpenes (over 60%) [7,9].

In the literature, there are current reports on the anticancer properties of several essential oils [23,26,30,31]. These active EO constituents appear to act synergistically with conventional chemotherapy and radiotherapy, and some clinical studies in humans have been initiated [23]. Anticancer activity has been detected in the EO of various plant species, e.g., *Achillea fragrantissima* [30], *Lycopus lucidus* [26], *Porcelia macrocarpa* [46], and neotropical piper species [31]. As shown in our study, the anticancer activities were also found in the EO of *A. montana* achenes (Figure 2, Figure 3 and Figure 4. Therefore, this plant species can be included in the group with high anticancer potential.

The chemical composition of EOs is determined by a wide range of factors. These include environmental conditions, seasonal variations, weather and climatic conditions, and plant development phases and age [6,14,15,47,48,49,50]. In turn, the effect of EO with a specific chemical composition has been reported to vary depending on the cancer type. Yu et al. [26] indicated that the cytotoxicity of EO from *L. lucidus* against liver carcinoma and breast cancer cell lines was significantly stronger than against other cell lines. Similarly, da Silva et al. [51] showed varied cytotoxic activity of *Piper aleyreanum* oils on colon and melanoma cell lines. Lima et al. [52] demonstrated various levels of cytotoxic activity of *P. klotzschianum* oils inhibiting human hepatocellular carcinoma, human promyelocytic leukemia, and murine melanoma cell lines. In turn, a broad cytotoxicity spectrum was demonstrated in the case of *P. cernuum* oil against, e.g., murine melanoma, human melanoma, human cervical tumor, human myeloid leukemia, and human glioblastoma cells [53,54]. In the present study, the quality of the EO-3 and EO-4 was similar. 2,5-dimetoxy-p-cymene was the dominant component of the EO; moreover, the impact of the EO on the MOGGCCM and T98G cell lines (apoptosis, necrosis, and autophagy) was similar. This implies that the age of arnica plants does not affect the studied chemical composition and antitumor activity of its essential oil.

Apoptosis or autophagy are mechanisms responsible for induction of programmed cell death in malignant gliomas [21,27,28,55]. The concentration of EO plays a key role in the treatment of human cancer cell lines through inhibition of cell growth and has an impact on these mechanisms [26]. In the case of the studied MOGGCCM and T98G lines, the concentration of 0.5 μL/mL initiated apoptosis without induction of necrosis and autophagy. The concentration of 1 μL/mL caused apoptosis without induction of autophagy. Thus, the biological activities of the EO from *A. montana* achenes make it a source of molecules that can be exploited in medicine and pharmaceutical industry. The results are very promising in the perspective of further studies focused on isolation of the main components, application thereof in studies on the MOGGCCM and T98G lines, and analysis of the typical programmed cell death and its molecular mechanism.

Gliomas are aggressive brain tumors with very high resistance to chemotherapy [32]. Natural bioactive compounds may act in synergy with drugs in pharmacological applications [56,57]. Therefore, in studies of the effectiveness of different substances in elimination of human glioma cells through apoptosis and autophagy, an attempt was made to use quercetin in such combinations [25,27,28,29]. Quercetin is a flavonoid found in *A. montana* and *A. chamissonis* flower heads and characterized by different biological, pharmacological, and medical applications [24,58]. The study of the effectiveness of such combinations in induction of programmed cell death in human gliomas has shown that quercetin exerts a toxic effect on the astrocytoma MOGGCCM cell line, inducing necrosis rather than programmed cell death [24]. However, the EO from the arnica montana achenes induced apoptosis of the MOGGCCM cell line without induction of necrosis and autophagy. The increase in the EO concentration to 1 μL/mL resulted in an increase in the level of apoptosis to 25.7% and 29.0% and to 27.7% and 32.7% in the MOGGCCM and T98G cell lines, respectively (Figure 2, Figure 3 and Figure 4, which suggests that it is a more promising natural anticancer product than quercetin.

There are only a few studies on the effects of EO or their components on glioma cells. Those with the most spectacular effects include α-bisabolol, i.e., a nontoxic natural compound that strongly induces apoptosis in glioma cells [34]. Thymol has been shown to have an inhibitory effect on apoptosis and cell growth in DBTRG-05MG human glioblastoma [59]. β-elemene, which is a natural plant drug extracted from *Curcuma wenyujin*, has shown promising anticancer effects against a broad spectrum of tumors, also inducing apoptosis of glioblastoma cells [22,60]. However, there are no literature reports of the effect of mountain arnica extracts or EOs on the induction of apoptosis of glioblastoma cells, especially in the MOGGCCM and T98G cell lines. The present study has shown that EO has an impact on glioma cell death. This is an important finding, especially in the light of recent investigations showing that gliomas naturally resist apoptosis [61]. The analyzed arnica EOs are characterized by dominance of 2,5-dimetoxy-p-cymene in the range of 39.54–44.65%; hence, they are a rich source of this highly interesting molecule [62]. Essential oils with this compound as a major component have antibacterial, antifungal, and insecticidal properties [63,64,65]. 2,5-dimetoxy-p-cymene is a dominant component of EO *Eupatorium triplinerve* [62], *Bubonium imbricatum* [63], *Ayapana triplinervis* [66], *Pulicaria mauritanica* [67], *Limbarda crithmoides* [68], and *Laggera crispata* [69]. Therefore, it probably plays an essential role in the anticancer activity of EO in relation to the MOGGCCM and T98G cell lines. The induction of apoptosis at a level of 25.7–32.7% presented in this paper has evidenced the anticancer activity of EO. The information obtained in this study indicates a need for further studies on the anticancer effect of arnica EOs and 2,5-dimetoxy-p-cymene against gliomas and on elucidation of the molecular anticancer mechanisms of this molecule.

## 4. Materials and Methods

### 4.1. Plant Material

Arnica achenes were collected when they were completely ripe. The achenes originated from 3-year-old and 4-year-old plants growing on experimental fields at the University of Life Sciences in Lublin located in the eastern part of Poland, (51°31′25″ N; 22°45′04″ E) on grey-brown podsolic soil with a granulometric composition of heavy loamy sand.

### 4.2. Qualitative and Quantitative Analysis of Essential Oil

#### 4.2.1. Assay of the Essential Oil Content

The *A. montana* achenes were air-dried and not crumbled. Essential oils were obtained by hydrodistillation using a Deryng type apparatus, according to the procedure of the Polish Pharmacopoeia VI [70]. The distillation time was 3 h. For this purpose, 20.0 g of achenes and 400 mL water was used. The distillation time was 3 h. The analysis was carried out in five repetitions.

#### 4.2.2. GC-MS Analysis

The chromatographic analysis was performed according to procedures described previously [7,9]. The analysis was performed in triplicate. The essential oils were analyzed using a Varian 4000 GC–MS/MS system (Varian, Palo Alto, CA, USA). The compounds were separated on a 30 m × 0.25 mm × 0.25 μm VF–5 ms column (Varian, Palo Alto, CA, USA). The column temperature was increased from 50 °C to 250 °C at a rate of 4 °C/min; injector temperature 250 °C; split ratio 1:50; injection volume 5 μL. The MS parameters were as follows: EI mode, with ionization voltage 70 eV, ion source temperature, 200 °C; scan range, 40–870 Da.

#### 4.2.3. Qualitative and Quantitative Analysis

The qualitative analysis was carried out on the basis of MS spectra, which were compared with the spectra from the NIST library [43] and with data available in the literature [42,71]. The identity of the compounds was confirmed by their retention indices [41] taken from the literature [42,71] and our data for standards described previously [7,9]. The quantitative analysis was performed with the internal standard addition method (alkanes C_12_ and C_19_) according to procedures described previously [72].

### 4.3. Glioma Cells and Culture

#### 4.3.1. Cells and Culture Conditions

Human glioblastoma multiforme cells (T98G, European Collection of Cell Cultures) and human anaplastic astrocytoma cells (MOGGCCM, European Collection of Cell Cultures) were grown in a 3:1 mixture of Dulbecco’s Modified Eagle Medium (DMEM) and Ham’s nutrient mixture F-12 (Sigma, St. Louis, MO, USA) supplemented with 10% fetal bovine serum (Sigma), penicillin (100 units/mL) (Sigma), and streptomycin (100 μg/mL) (Sigma, St. Louis, MO, USA). The cultures were kept at 37 °C in a humidified atmosphere of 95% air and 5% CO_2_. Primary cultures of human skin fibroblast were prepared according to a method described previously [73].

#### 4.3.2. Detection of Apoptosis, Necrosis, and Autophagy

Apoptosis, autophagy, and necrosis were identified microscopically after staining with fluorochromes Hoechst 33342 (Sigma), acridine orange (Sigma, St. Louis, MO, USA), and propidium iodide (Sigma, St. Louis, MO, USA) respectively, as described previously [27,28,29]. A fluorescence microscope (Nikon E-800, Tokyo, Japan) was used for morphological analysis of dead cells. At least 1000 cells in randomly selected microscopic fields were counted under the microscope. Each experiment was repeated three times with each 1000 cells. In addition, 50% inhibitory concentrations (IC50 values) were determined for all the tested extracts using GraphPad Prism version 7 (GraphPad Software, San Diego, CA, USA).

### 4.4. Statistical Analysis

The normality (Shapiro-Wilk test) and variance heterogeneity (Levene test) were tested and transformed data were used when necessary. The two-way analysis of variance (ANOVA) and subsequent Tukey tests were used. The results were expressed as means ± SD, and the differences were considered significant at *p* < 0.05. The statistical analyses were carried out using the Statistica 6.0 software (Stat. Soft, Inc., Kraków, Polska).

## 5. Conclusions

The essential oil from *A. monatana* achenes is described in this paper for the first time. Its quality and chemical composition are different from those of essential oils from flower heads and roots of arnica populations in different regions of Europe. EOs exert an anticancer effect by induction of anaplastic astrocytoma and glioblastoma multiforme cell death. The activity expressed by induction of apoptosis at a level of 25.7–32.7% facilitates the use of this secondary metabolite in subsequent studies focused on the development of glioma therapy in the future. The essential oil from *A. monatana* achenes is characterized by dominance of 2,5-dimetoxy-p-cymene. Probably, this component plays a key role in the anticancer activity against MOGGCCM and T98G cell lines. The knowledge and information obtained in this study indicate a need for further research on the anticancer effect of 2,5-dimetoxy-p-cymene on the MOGGCCM and T98G cell lines and for elucidation of the molecular anticancer mechanisms of this compound.

## Figures and Tables

**Table 1:** Composition of essential oil from *A. montana* achenes collected from 3-year-old (EO-3) and 4-year-old (EO-4) plants.

***A. montana* Achenes**				EO-3	EO-4
				Essential Oil Content [% v/w] ± SD	
				0.167 ± 0.015	0.145 ± 0.012
**Compound**	**RI**	**RI _LIT_**	Identification	Essential Oil Composition [%] ± SD	
Cumene	928	924	MS, RI	13.24 ± 0.12	10.71 ± 0.04
1,2,2,3-Tetramethylcyclopent-3-Enol	1034	1030 ^a^	MS, RI	4.33 ± 0.31	2.94 ± 0.15
α-Pinene Oxide	1105	1099	MS, RI	1.87 ± 0.24	1.15 ± 0.11
Borneol	1176	1165	MS, RI	0.58 ± 0.39	0.47 ± 0.06
Decanal	1210	1201	MS, RI ^e^	7.31 ± 0.82	6.28 ± 0.50
Thymol Methyl Ether	1234	1232	MS, RI	8.66 ± 0.46	8.63 ± 0.19
Presilphiperphol-7-Ene	1339	1334	MS, RI	0.49 ± 0.07	0.55 ± 0.02
7-Epi-Silphiperfol-5-Ene	1350	1345	MS, RI	0.74 ± 0.01	0.82 ± 0.08
β-Maaliene	1395	1389 ^b^	MS, RI	0.88 ± 0.11	0.39 ± 0.04
α-Isocomene	1391	1387	MS, RI	3.49 ± 0.12	4.16 ± 0.13
2,5-Dimetoxy-p-Cymene	1429	1424 ^c^	MS, RI	39.54 ± 0.74	44.65 ± 0.61
E-Caryophyllene	1423	1417	MS, RI ^e^	1.12 ± 0.07	1.40 ± 0.06
E-α-Bergamotene	1441	1432	MS, RI	0.85 ± 0.01	0.73 ± 0.08
2,6-Diisopropylanisole	1444	1438	MS, RI	8.55 ± 0.21	8.41 ± 0.04
Lippifoli-1(6)-En-5-One	1560	1550	MS, RI	1.40 ± 0.14	0.46 ± 0.01
Caryophyllene Oxide	1588	1582	MS, RI ^e^	1.09 ± 0.10	1.54 ± 0.09
2-Pentadecanone-6,10,14-Trimethyl	1849	1846	MS, RI	-	2.22 ± 0.26
β-Springene	1921	1918 ^a,d^	MS, RI	-	0.87 ± 0.06
Monoterpenes				23.77	20.49
Aliphatic Aldehydes				7.31	6.28
Sesquiterpenes				10.06	10.05
Phenyl Derivative, Ether				48.09	53.06
Others				4.91	6.5
Sum of Identified (%)				94.14	96.38

RI—retention indices (from temperature-programming, using definition of Van Den Dool and Kratz [41]). RI_Lit_—retention indices taken from literature [42], ^a^ [43], ^b^ [7], ^c^ [4], ^d^ [44] ^e^ identified based on comparison with standards.

**Table 2:** IC50 value for the essential oil from mountain arnica achenes collected from 3-year-old (EO-3) and 4-year-old (EO-4) plants.

Cell Line	EO-3	EO-4
MOGGCCM	1.6	1.8
T98G	2.1	2.0
